# Antiretroviral Drug Resistance and Routine Therapy, Cameroon

**DOI:** 10.3201/eid1206.050860

**Published:** 2006-06

**Authors:** Christian Laurent, Charles Kouanfack, Laurence Vergne, Michèle Tardy, Léopold Zekeng, Nathalie Noumsi, Christelle Butel, Anke Bourgeois, Eitel Mpoudi-Ngolé, Sinata Koulla-Shiro, Martine Peeters, Eric Delaporte

**Affiliations:** * Institut de Recherche pour le Développement (UMR 145), Montpellier, France;; † Central Hospital, Yaoundé, Cameroon;; ‡ Laboratoire de Santé et d'Hygiène Mobile, Yaoundé, Cameroon;; § National AIDS Program, Yaoundé, Cameroon;; ¶ Military Hospital, Yaoundé, Cameroon

**Keywords:** HIV, antiretroviral, resistance, routine care setting, Africa, dispatch

## Abstract

Among 128 patients routinely receiving highly active antiretroviral therapy in an HIV/AIDS outpatient clinic in Cameroon, 16.4% had drug resistance after a median of 10 months. Of these, 12.5% had resistance to nucleoside reverse transcriptase inhibitors (NRTIs), 10.2% to non-NRTIs, and 2.3% to protease inhibitors.

HIV drug resistance is a major threat to the scaling up of antiretroviral therapy (ART) in developing countries (the World Health Organization/United Nations Programme on HIV/AIDS "3 by 5" Initiative) (*1*), especially in Africa (*2*). Inadequate clinical and biological follow-up has been linked to high rates of drug resistance (>50% after 8 to 20 months) in Gabon (*3*), Côte d'Ivoire (*4*), and Uganda (*5*). In a recent study in public and private health care clinics in Douala, the economic capital of Cameroon, we found that the clinical and biological follow-up and drug supply were irregular and that many patients interrupted their treatment (*6*). Data on drug resistance in the routine care setting are urgently required to design large, effective ART programs. We describe the frequency and nature of major genotypic mutations conferring resistance to antiretroviral drugs among patients treated in a routine HIV/AIDS outpatient clinic in Yaoundé, the political capital of Cameroon.

## The Study

We conducted a cross-sectional survey from January 2002 to January 2004 among HIV-1–infected patients managed at the Central Hospital. The patients had to pay for their drugs (US $23–$100 monthly) and laboratory tests (US $58–$85 per viral load assay and $19–$27 per CD4 cell count). Consequently, follow-up was often irregular. All patients who were given ART for at least 3 months were eligible for the study. Approximately 15%–20% of eligible patients refused or were not asked (physicians forgot) to participate. Blood samples were not available for 9 other patients. The Cameroon national ethics committee approved the study protocol, and patients gave their informed consent. Basic demographic and medical data were recorded on a standard questionnaire.

HIV was typed in each patient (HIV-1 group M, N, or O, or HIV-2) with an in-house enzyme-linked immunosorbent assay (ELISA) based on V3 loop peptides (*7*). Genotypic resistance to antiretroviral drugs was studied by sequencing the protease and reverse transcriptase genes with group M- or O-specific primers, depending on the serotyping results (*8*); samples that could not be typed with ELISA were tested with both group M and O primers. Briefly, viral RNA was extracted from plasma with the QIAamp Viral RNA minikit (Qiagen, Courtaboeuf, France) and reverse transcribed to cDNA by using Expand RT (Boehringer, Mannheim, Germany) and a reverse primer. An 1,800-bp fragment encompassing the protease and reverse transcriptase genes was amplified by nested polymerase chain reaction and directly sequenced with an ABI PrISM Big Dye Terminator cycle sequencing ready reaction kit (Perkin-Elmer, Roissy, France). Genetic subtypes were determined by phylogenetic tree analysis with the Clustal W program (*8*). The deduced amino acid sequences were compared with a reference sequence to detect mutations associated with resistance. Mutations were classified as minor or major, by using the September 2004 version of the French National Agency for Research on AIDS consensus statements on antiretroviral drug resistance (http://www.hivfrenchresistance.org). A susceptible strain based on absence of major drug resistance mutations by genotyping or a strain that could not be amplified for genotyping was considered nonresistant.

One hundred twenty-eight HIV-1–infected patients received ART for a median of 10 months (interquartile range [IQR] 7–18). Median age was 39 years (IQR 33–46); 70 (54.7%) of the patients were women. In addition to nucleoside reverse transcriptase inhibitors (NRTIs), 94 patients (73.4%) had received non-NRTIs (59 patients received only efavirenz, 30 received only nevirapine, and 5 received both) and 53 patients (41.4%) had received protease inhibitors (PIs, 50 patients received only indinavir, 2 received only nelfinavir, and l received both); 19 patients had received both non-NRTIs and PIs. Two patients (1.6%) initially received only 2 NRTIs (lamivudine and zidovudine for 7 months in 1 case; stavudine and didanosine for 14 months in the other). Samples from 113 patients (88.3%) reacted with group M peptides, 3 samples (2.3%) reacted with group O peptides, and 2 other samples (1.6%) reacted with both group M and O peptides. Ten samples did not react with group M, N, or O or HIV-2 peptides. Thirty-five samples could be amplified, and all were characterized in the pol gene. The circulating recombinant form (CRF) 02-AG strain predominated (22 patients, 62.9%); the other 13 patients had subtype A (1), D (2) or F2 (3), or CRF01-AE (2), CRF02-AG/F (2), CRF11-cpx (2), or CRF13-cpx (1).

Major genotypic mutations associated with antiretroviral drugs resistance were detected in 21 patients (16.4%, 95% confidence interval 10.5–24.0). The characteristics of these patients are shown in the Table. Sixteen patients (12.5%) had resistance to NRTIs (Figure) due to the mutations M184V (15 patients), M184I (1), T215Y (1), T215F (3), K65R (2), and Q151M (1); thymidine analog mutations M41L (2), D67N (2), K70R (3), K219Q (1), and K219E (1) were also detected. Thirteen patients (10.2%) had resistance to non-NRTIs due to the mutations K103N (11), K101E (1), Y181C (1), Y188L (2), G190E (1), and P225H (2). Three patients (2.3%) had resistance to PIs due to the mutations V82A (2 patients) and N88D (1). The 2 patients treated for a time with only 2 NRTIs (patients 2-59 and 2-84, Table) had several major genotypic mutations but had received ART for 52 and 48 months, respectively.

**Figure:**
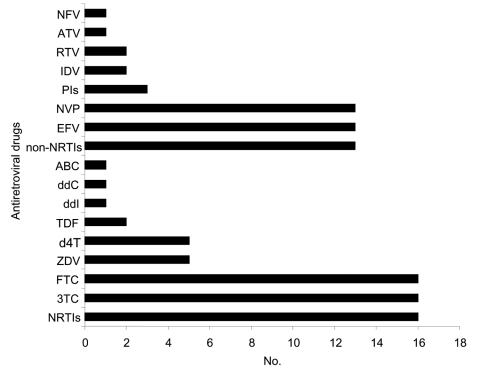
Patients with resistance to antiretroviral drugs. NFV, nelfinavir; ATV, atazanavir; RTV, ritonavir; IDV, indinavir; PIs, protease inhibitors; NVP, nevirapine; EFV, efavirenz; non-NRTIs, non-nucleoside reverse transcriptase inhibitors; ABC, abacavir; ddC, zalcitabine; ddI, didanosine; TDF, tenofovir; d4T, stavudine; ZDV, zidovudine; FTC, emtricitabine; 3TC, lamivudine; NRTIs, nucleoside reverse transcriptase inhibitors.

**Table:** Antiretroviral drug resistance in 21 patients receiving multiple ART in a routine care setting in Cameroon*

Patient no.	Age	Sex	Antiretroviral drugs received	Months from start of ART	Drug resistance	Major genotypic mutations	Subtype pol
2-29	46	F	3TC, ZDV, EFV	33	3TC, FTC, EFV, NVP	M184V, K103N, P225H	CRF02-AG
2-44	49	F	3TC, ZDV, EFV	10	3TC, FTC, EFV, NVP	M184V, (K70R), K103N, Y188L	CRF02-AG
2-47	42	M	3TC, ZDV, IDV	10	3TC, FTC	M184V	CRF02-AG
2-59	36	F	3TC, ZDV, EFV, IDV	52	3TC, ZDV, d4T, FTC, EFV, NVP	M184V, T215F, (M41L), K103N	CRF02-AG
2-66	36	M	3TC, ZDV, d4T, ddI, EFV	21	3TC, FTC, EFV, NVP	M184V, K103N	CRF02-AG
2-70	30	M	d4T, ddI, EFV	6	EFV, NVP	K103N	CRF02-AG/F
2-75	37	F	3TC, d4T, IDV	9	3TC, FTC	M184V	A
2-76	34	M	3TC, d4T, EFV	10	3TC, ZDV, d4T, FTC, EFV, NVP	M184V, T215Y, K103N	F2
2-84	51	M	3TC, ZDV, d4T, ddI, NFV	48	NRTIs, NFV	K65R, M184V, Q151M, N88D	D
2-91	44	M	3TC, d4T, EFV, IDV	6	EFV, NVP	G190E	CRF02-AG/F
2-98	32	F	d4T, ddI, IDV	7	IDV, RTV	V82A	D
22-2	42	M	3TC, ZDV, EFV	14	3TC, FTC, EFV, NVP	M184I, (M41L), K101E, K103N	CRF02-AG
22-9	33	F	3TC, ZDV, d4T, ddI, EFV, IDV	31	3TC, FTC, EFV, NVP	M184V, K103N, P225H	CRF11-cpx
22-25	30	F	3TC, ZDV, EFV, IDV	18	3TC, FTC	M184V	CRF02-AG
22-31	42	M	3TC, d4T, IDV	8	3TC, FTC	M184V	CRF02-AG
22-33	41	F	3TC, ZDV, IDV	18	3TC, FTC	M184V	CRF02-AG
22-35	58	M	3TC, ZDV, IDV	17	ATV, IDV, RTV	V82A	CRF01-AE
22-47A	48	F	3TC, ZDV, EFV, IDV	45	3TC, ZDV, d4T, FTC, EFV, NVP	M184V, T215F, (D67N, K70R, K219Q), K103N, Y188L	CRF02-AG
22-50	32	M	3TC, d4T, NVP	6	3TC, FTC, TDF, (ABC, ddI), EFV, NVP	K65R, M184V, Y181C	CRF01-AE
22-57	53	M	3TC, ZDV, EFV	7	EFV, NVP	K103N	CRF02-AG
22I-75	50	F	3TC, ZDV, d4T, ddI, EFV, IDV	29	3TC, ZDV, d4T, FTC, EFV, NVP	M184V, T215F, (D67N, K70R, K219E), K103N	CRF02-AG

*ART, antiretroviral therapy; 3TC, lamivudine; ZDV, zidovudine; EFV, efavirenz; FTC, emtricitabine; NVP, nevirapine; IDV, indinavir; d4T, stavudine; ddI, didanosine; NFV, nelfinavir; NRTIs, nucleoside reverse transcriptase inhibitors; ATV, atazanavir; RTV, ritonavir; TDF, tenofovir; SQV, saquinavir; ABC, abacavir. Resistances in parentheses indicate possible resistances. Mutations in parentheses indicate thymidine analogue mutations.

## Conclusions

This observational study showed that 16.4% of patients receiving ART in a routine care setting in Cameroon had drug resistance after a median of 10 months. The rate of resistance was lower than that observed in earlier studies in Côte d'Ivoire (*4*), Gabon (*3*), and Uganda (*5*). Several factors could explain this finding. First, a history of suboptimal therapy was rare: only 2 patients had received a 2-drug regimen, and none had received single-agent therapy. Second, 90% of our patients began receiving ART after a national consensus conference held in June 2000 had standardized the drugs supply, drugs regimen, and clinical and biological follow-up. Third, the physicians were trained and experienced in ART use. Fourth, the cost of drugs and laboratory tests has fallen in recent years in Cameroon, a fact that favors adherence to therapy. Our methods could also account for the difference: our median follow-up period was substantially less than that in the studies in Gabon and Uganda, so that our patients had less time for resistance to develop, and our assumption that nonamplification was equivalent to nonresistance could have led to an undercount of resistant strains. Lower rates of resistance were achieved in pilot studies in Cameroon (*9*) and Senegal (*10**,**11*), thanks to measures favoring adherence to therapy, such as provision of drugs and laboratory follow-up at no cost (or for a limited charge), and psychosocial support (counseling, access to discussion groups, and active search for patients who did not attend scheduled clinical visits, biological examinations, or drug dispensing sessions).

Resistance most often involved lamivudine (12.5%; and emtricitabine, due to mutation M184V/I related to lamivudine pressure [emtricitabine was not used in Cameroon]), efavirenz, and nevirapine (10.2%). These drugs are widely used in Cameroon in either individual formulations or fixed-dose combinations (lamivudine/zidovudine, lamivudine/stavudine, lamivudine/stavudine/nevirapine, and lamivudine/zidovudine/nevirapine). The fixed-dose combination of lamivudine/stavudine/nevirapine is now the most frequently prescribed drug in Cameroon and other African countries (*12*). In our study, 19 patients (14.8%) had resistance to >1 component on this fixed-dose combination, and high rates of resistance could compromise the use of this inexpensive (US $4.5 monthly) and convenient drug. Frequent resistance to nevirapine could also compromise the use of this drug for preventing mother-child transmission (most such programs in Africa, including in Cameroon, are based on nevirapine).

Our study showed a relatively low level of resistance after a median duration of 10 months' treatment in a routine care setting, but we could not evaluate the association of resistance with adherence, support, or prescribing practices. The differences in methods among the African cross-sectional studies of resistance, including our own and the others referenced, make comparisons among countries difficult, although some differences are likely due to prescribing practices, drug availability, support for adherence, and follow-up. More extensive prospective studies that use standardized methods could provide comparable estimates of resistance seen at specific times (e.g., 6, 12, and 24 months after ART begins) in different countries and delineate ART program factors associated with a low prevalence of resistance.
